# Intra-Abdominal Malignant Melanoma: Challenging Aspects of Epidemiology, Clinical and Paraclinical Diagnosis and Optimal Treatment—A Literature Review

**DOI:** 10.3390/diagnostics12092054

**Published:** 2022-08-24

**Authors:** Sinziana Ionescu, Alin Codrut Nicolescu, Octavia-Luciana Madge, Laurentiu Simion, Marian Marincas, Mihai Ceausu

**Affiliations:** 1 General Surgery and Surgical Oncology Clinic I of the Bucharest Oncology Institute, “Carol Davila” University of Medicine and Pharmacy, 022328 Bucharest, Romania; 2 Roma Medical Center for Diagnosis and Treatment, 011774 Bucharest, Romania; 3 General Surgery and Surgical Oncology Clinic I of the Bucharest Oncology Institute, University of Bucharest, 022328 Bucharest, Romania; 4 Pathology Department of the Bucharest Oncology Institute, “Carol Davila” University of Medicine and Pharmacy, 022328 Bucharest, Romania

**Keywords:** malignant melanoma, abdominal metastases, diagnosis, review, surgical oncology, dermato-oncology, immunotherapy, surgery, prognosis, achromic melanoma

## Abstract

According to European consensus-based interdisciplinary guidelines for melanoma, cutaneous melanoma (CM) is the most deadly form of dermatological malignancy, accounting for 90% of the deaths of skin cancer patients. In addition to cutaneous melanoma, mucosal melanoma occurs in four major anatomical sites, including the upper respiratory tract, the conjunctiva, the anorectal region, and the urogenital area. As this cancer type metastasizes, a classification used in the current medical literature is the distinction between secondary lesions and primary malignant melanoma of the abdominal cavity. Given that malignant melanoma is the most common cancer that spreads to the gastrointestinal tract, different imaging modalities compete to diagnose the phenomenon correctly and to measure its extension. Treatment is primarily surgery-based, supported by immunotherapy, and prolongs survival, even when performed at stage IV illness. In the end, special forms of malignant melanoma are discussed, such as melanoma of the genito-urinary tract and amelanotic/achromic melanoma. The importance of this present literature review relies on yielding and grouping consistent and relevant, updated information on the many aspects and challenges that a clinician might encounter during the diagnosis and treatment of a patient with intra-abdominal melanoma.

## 1. Introduction

**Figure 1:**
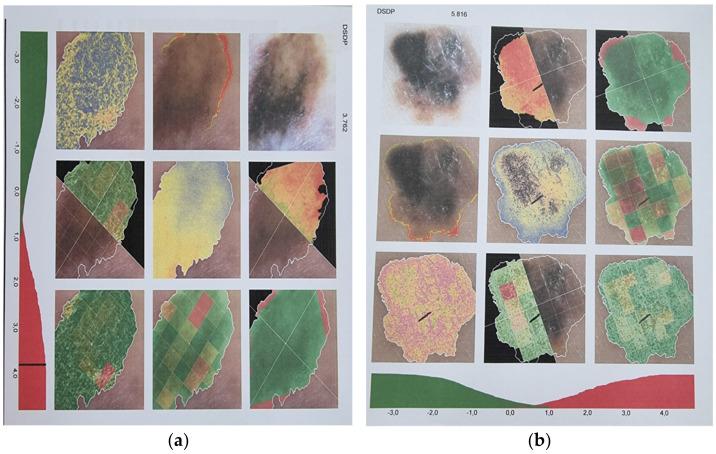
Cutaneous malignant melanomas (**a**,**b**), as seen with the help of digital dermatoscopy, illustrating aspects of polarized light, color, form, margins, number of colors, color homogeneity, number of structures, structure asymmetry, and structure homogeneity.

According to the European consensus-based interdisciplinary guidelines on melanoma [1], cutaneous melanoma (CM) is the most lethal form of skin tumor and is responsible for 90% of skin cancer deaths. As demonstrated in Figure 1a,b, melanoma can be identified clinically and should always be confirmed by dermatoscopy.

When melanoma is suspected, histopathological examination is vital and always required. According to the ninth version of the American Joint Committee on Cancer classification, melanomas are classified and described. Up to 0.8 mm in thickness, thin melanomas do not require additional imaging examination. Sonography of lymph nodes is recommended for examinations beginning at stage IB; no additional imaging procedures are recommended. In addition to brain magnetic resonance imaging, whole-body computed tomography (CT) or positron emission tomography-computed tomography (PET-CT) scans are indicated beginning at stage IIC. At stages III and above, mutation testing is recommended, particularly for the BRAF V600 mutant.

**Figure 2:**
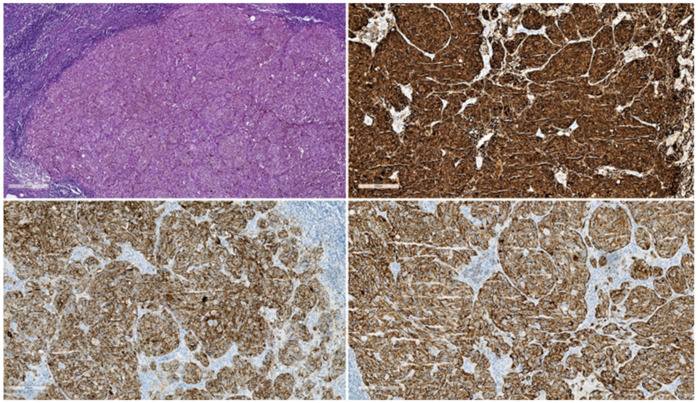
Secondary metastases of cutaneous melanoma in a mesenteric lymph node: Left up panel: Solid mass of tumor cells without pigment deposition, HE, 50× Right up panel: diffuse strong positive reaction to S-100 in tumor cells, left down panel: diffuse positive reaction to HMB-45, IHC, 100×, right down panel: diffuse positive reaction to MART-1 in tumor cells, IHC, 100×. The existence or absence of nodal metastases in melanoma has long been acknowledged as a crucial aspect of patient care. However, there has been a lot of discussion over the specifics of that relevance. Researchers and clinicians [5] have split into two groups: those who think lymph nodes are an incubator for sequential progression and metastasis, and others who think lymph nodes are just a marker of any melanoma’s potential for spread. In terms of managing localized nodes when there are no clinically obvious symptoms at the time of diagnosis, the disagreement has had a considerable practical impact on therapy.

Intra-abdominal melanoma can manifest either as a metastatic lesion from another anatomic site or as a new lesion; in both cases, the lesion might present clinically as an emergency. Metastatic cutaneous melanoma typically affects the liver and pelvic lymph nodes (Figure 2). Lower extremities’ primary tumor rates are among the highest (52 percent). In addition, a structured follow-up is crucial for identifying relapses and secondary primary melanomas as soon as possible. Apart from cutaneous melanoma, mucosal melanoma develops at four major anatomical sites [2], including the upper aerodigestive tract, the conjunctiva, the anorectal and urogenital tracts, and of course, it can also present with metastatic spread.

It has been shown [3] that both cutaneous and mucosal melanomas contain melanocytes, yet there are considerable differences in their prognosis, localization, age at presentation, appearance, and exposure to ultraviolet radiation (clearly associated with cutaneous melanoma and poor prognosis in the case of MM). Metastatic illness in stage IV has effective therapeutic options, including BRAF-targeted therapy [4]. Furthermore, the best treatment (which concerns overall survival) indicated in symptomatic localized disorders that are unresponsive to conventional medical interventions still heavily rely on surgery.

## 2. Materials and Methods

The purpose of the present article is a well-documented literature review on intra-abdominal malignant melanoma. In order to achieve this goal, an extensive research on several databases was performed between 3 May 2022 and 3 June 2022, as follows: (1) on www.scopus.com, the terms: ”intra-abdominal metastases from malignant melanoma”, with the filters “medicine”, “review”, “English” retrieved 260 documents; (2) another search on www.scopus.com, for the terms: ”intra-abdominal malignant melanoma”, with the filters: 2018–2022, subject area “medicine”, “English”, retrieved 207 results; (3) a third search in the same database was done for the terms ”melanoma of unknown primary”, with the filter “after 2015” and “abdominal AND melanoma AND systematic AND review”, limited to English, medicine and journal; (4) a fourth search was done on www.scopus.com, looking for: “melanoma of the urinary tract review”, with filters: medicine, 2017–2022, English, Journals, reviews found 107 searches and the number of systematic reviews was 68; (5) on the www.pubmed.org the terms were: “intra-abdominal malignant melanoma” with filters English and humans and the findings counted 67 articles; (6) another search query on www.sciencedirect.com, with the filters “subscribed journals”, 2017–2022, review articles, medicine and dentistry retrieved 263 findings; (7) also in the Sciencedirect platform database, the terms searched for were “amelanotic melanoma of the abdomen”, with the filters “subscribed journal”, 2017–2022, “medicine and dentistry” and the quest returned 40 results; (8) the terms “achromic AND melanoma AND metastases” were also searched for on sciencedirect and, moreover, (9) on ScienceDirect, Pubmed and Oxford Academy journals databases the search “(urinary OR kidney OR bladder OR urethra OR ureter)AND melanoma” was also conducted; (10) on Oxford Academic Journals database the search for “intra-abdominal malignant melanoma” with the filters “journal article”, 2017–2022, medicine and health finally retrieved 155 results. The present article, treating the subject of intra-abdominal malignant melanoma, is based on the results of the above-mentioned searches grouped into categories according to the issues debated in the literature. Table 1 illustrates the research sites/databases in correlation with the search terms and the additional filters employed in the literature quest for data on intra-abdominal melanoma.

## 3. Main Findings of the Literature (Review) Quest

In the present medical literature, differentiation between intra-abdominal primary malignant melanomas and secondary lesions with the same site is one of the classifications considered. All clinical research begins from the fundamental and is completed with vital information from animal models. Those provide various and extensive chances for study of different medical and surgical aspects, with careful adherence to bioethical laws and regulations.

### 3.1. Experimental Animal Models

Despite being the most prevalent malignant primary ocular tumor in adults, uveal melanoma (UM) is a rare form of melanoma. Nearly fifty percent of individuals with primary UM develop liver-based systemic metastases. There is currently no viable treatment for UM hepatic metastases, and the outcome is invariably bleak. Developing a treatment plan for UM hepatic metastases is impeded by the lack of adequate animal models. The liver tumor genesis using two orthotopic mouse models for human UM hepatic metastases was investigated [6], with two main models found: direct hepatic implantation model (intrahepatic dissemination model) and splenic-implantation model (hematogenous dissemination model). For the development of in vivo tumor models, a human UM cell line derived from hepatic metastasis and nonobese diabetic severe combined immunodeficient mice was utilized. In every instance of the direct hepatic implantation model, a localized tumor grew in the liver, followed by around fifty percent intrahepatic spread. After splenic implantation, numerous hepatic metastases were identified in the splenic implantation model. Subsequently, hepatic tumors gave rise to intra-abdominal metastases, but no lung metastases were observed. These results align with those reported in human UM liver metastases. These orthotopic mouse models are useful for examining the biological activity of human UM liver cells. Additional information on the study of malignant melanoma in animal models can be found in Table 2.

### 3.2. Primary Intra-Abdominal Melanoma

#### 3.2.1. Originating in the Gastrointestinal Tract

Malignant gastrointestinal melanoma is frequently a metastatic lesion. The lymphatic route is the predominant mode of transmission, while the hematogenous route is secondary. Most gastrointestinal metastases consist of intraluminal mucosal melanomas. In contrast, primary malignant melanoma developing in the digestive tract is extremely rare, with the anorectum and oral cavity being the most commonly affected sites.

##### From the Esophagus

Primitive malignant melanoma of the esophagus has primarily been covered in case reports [16]. This tumor is believed to account for between 0.1 and 0.2% of all esophageal cancers and has a disappointing prognosis. There have been reports of recurrence after the initial resection and the necessity for intervention, as well as a modest survival rate after the original diagnosis.

##### From the Stomach

In a systematic review [17], it was found that the principal symptoms included abdominal pain (64%), weight loss (48%), and hematemesis or melena (hematemesis or diarrhea) (32%). The most common tumor location was the body of the stomach (54.2%). All tumors were removed surgically, the median recurrence time was 5 months, and 12% of patients reached the 5-year survival milestone. Primary gastric melanoma is a disease characterized by aggressive malignant behavior and it is crucial to distinguish this condition from a metastatic lesion. A prompt diagnosis and treatment strategy are required.

##### From the Colon

In research from 2018 [18], it is mentioned that malignant melanomas having the colon as a starting point are only scarcely encountered (less than 40 cases reported), and that, when histopathological analysis of the surgical specimen suggested malignant melanoma, immunohistochemical analysis confirmed it with testing for the S100 protein, Melan-A, HMB-45, and vimentin. A series of postoperative clinical, laboratory, and imaging examinations revealed the absence of any suspicious lesions in the skin, eye, leptomeninges, or other sites. The diagnosis of primary colonic melanoma was therefore confirmed, mostly thorough exclusion of other likely causes. These rare tumors are difficult to diagnose and typically necessitate a multidisciplinary treatment approach, including surgery, chemotherapy, immunotherapy, and possibly, radiotherapy.

##### From the Pancreas

Various research has described that metastatic melanoma from an occult primary rarely occurs; as opposed to this, unknown primary melanoma in the pancreas is even prevalent. However, it is biologically undefined and clinically understudied [19].

##### From the Ovary

The first reference of ovarian melanoma in primigravida has also been reported [20]. Further research shows [21] that about one-third of diagnosed melanoma patients are of childbearing age. The annual incidence of melanoma has increased steadily over the past four decades, resulting in a rise in the number of pregnant and postpartum women diagnosed with the disease. There are currently no formal guidelines for the early and metastatic management of pregnancy-associated melanoma (PAM).

To evaluate the clinical manifestation, treatment options, and prognosis of primary melanomas arising from ovarian cystic teratomas (OCT), various studies [22] have concluded that OCT-originating malignant melanoma is a rarely encountered disease with a modest outcome.

In research published in 2021 [23], the significance of establishing an international database of rare ovarian tumors was underlined, as it would enable the collection of data from various oncological centers and further study of these tumors.

##### From the Anorectal Region

Multiple hepatic metastases are rarely the initial manifestation of primary anal malignant melanoma [24]. The authors further reported FDG PET/CT findings of pathology-confirmed hepatic metastases from anal malignant melanoma of unknown origin in a 43-year-old woman with worsening abdominal pain at presentation.

##### From the Gallbladder

The primary malignant melanoma of the gallbladder is referred to as an “outstandingly rare” tumor [25].

##### From the Small Intestine

A primary small bowel melanoma is a very uncommon tumor [26] and a definitive diagnosis cannot be made until a comprehensive investigation has been conducted to rule out the presence of a primary lesion. After an analysis on 36 cases from the literature, [27] it was concluded that primary small bowel MM appears to be a very rare condition that clinicians should be more aware of in order to plan a more accurate strategy for early diagnosis and treatment.

##### From the Adrenals

Primary adrenal melanoma (PAM) was an extremely rare condition, as evidenced by the small number of cases described in the medical literature. In a case presentation [28], a 58-year-old man was admitted to the hospital with intermittent left flank pain that lasted a month. The renal computed tomography (CT) scan revealed that a large (15.5 cm/12.1 cm/13.0 cm) retroperitoneal tumor appeared to originate from the left adrenal gland. The patient was diagnosed with PAM based on: clinical symptoms, previous history, physical examination, and postoperative pathology. The patient’s retroperitoneal tumor was removed through an open surgical procedure. The patient participated in a clinical drug trial and received ipilimumab as adjuvant medical therapy following surgery.

#### 3.2.2. Association with Extracutaneous Blue Naevi

Blue naevi are presented [29] as rare dermal melanocytic neoplasms characterized by GNAQ/GNA11 mutations, which very infrequently progress to melanoma. These melanomas typically have BAP1 mutations and lack the typical genetic alterations of conventional melanoma. Blue nevi occasionally arise at extracutaneous sites. The study concluded that aggressive melanomas emerging in extracutaneous blue naevi must be separated from metastatic melanoma, gastrointestinal stromal tumor, and malignant melanotic nerve sheath tumor due to the major treatment and prognosis disparities among these tumor forms. In a retrospective research conducted by [30] on pediatric melanoma, of the 38 identified cases of fatal pediatric melanoma, 57% were diagnosed in white patients and 19% in Hispanic patients. The median age at diagnosis was 12.7 years, and the median age at death was 15.6 years. 50 percent of the cases with known subtypes were nodular (8/16), 31 percent had superficial spread (5/16), and 19 percent were spitzoid (3/16). Ten percent (10/38) of melanomas were associated with congenital melanocytic nevus.

### 3.3. Secondary Intra-Abdominal Melanoma

#### 3.3.1. Primary Intraocular Melanoma and Its Metastases

Cutaneous melanoma and uveal melanoma are biologically different tumors [31]. In uveal melanoma, GNAQ/GNA11 mutations are prevalent, whereas BRAF, PTEN, TP53, and CDKN2A mutations are prevalent in cutaneous melanoma. The treatment of primary intraocular uveal melanoma has improved greatly, its driver genes have been deciphered in large part, and the methods for estimating its risk for metastasis, which are based on an international staging system and genetic data, are highly accurate. The risk of acquiring distant metastases, which affects nearly half of all patients, remains unchanged. Metastases are the major cause of death following a uveal melanoma diagnosis, yet there is no consensus about surveillance, staging, and treatment of disseminated disease, and survival rates have not improved until recently. It has been reported [32] that up to fifty percent of patients develop metastatic disease, with the liver being the most common secondary determination. Patients at this metastatic stage present with a poor survival rate (4 to 15 months), and this aspect has not changed significantly in the past several years. The final frontier in the conquest of uveal melanoma is the resolution of these problems in order to treat metastatic disease [33]. Crucial steps toward the final frontier of curing metastatic uveal melanoma could well be represented by: inducing dormancy of the micro-metastasis, harmonizing surveillance protocols, promoting staging, identifying predictive factors, initiating controlled clinical trials, and standardization of the reported results.

#### 3.3.2. Cutaneous Malignant Melanoma and Its Metastases

Malignant melanoma is the most prevalent cutaneous malignancy to spread to the digestive (GI) tract [34]. It has a predilection for spread to the small intestine, followed by involvement of the stomach and of the large bowel. For a precise diagnosis of GI involvement by a metastatic MM, there are excellent endoscopic options, including video capsule endoscopy and enteroscopy.

##### Clinical Aspects

The primary tumor and the moment of the appearance of metastases can be temporarily spaced: [35] case of a nasal melanoma that metastasized to the pancreas 10 years after the diagnosis of the primary has been described, in addition to reports [36] of a similar phenomenon for choroidal melanoma. Furthermore, the differential diagnosis of a solid pancreatic mass should include metastatic melanoma [37], as shown in a previous section.

##### Paraclinical Aspects

In a study from 1982 [38], the sonographic appearance of the metastatic malignant melanoma was described, and the sonographic findings of 42 patients with intra-abdominal malignant melanoma metastases were presented. Ten of the 42 patients had multiple metastatic sites, with the liver being the most commonly affected organ. The majority of liver metastases had low echo amplitude, and fluid was visible within 30% of all metastases, likely indicating the frequency of hemorrhage into these lesions.

In 1988 [39], in 88 patients with pathologically confirmed cutaneous melanoma, a retrospective evaluation of radionuclide liver and spleen scintigraphy (LS), ultrasonography (US), and computed tomography (CT) was performed. CT was found to detect metastases significantly earlier than US when compared to US. Overall, CT was the most accurate method for the detection of intra-abdominal cutaneous melanoma metastases.

**Figure 3:**
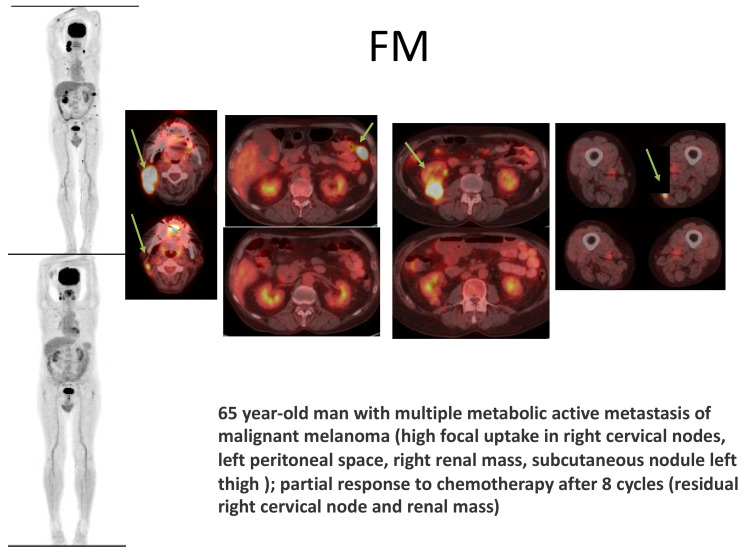

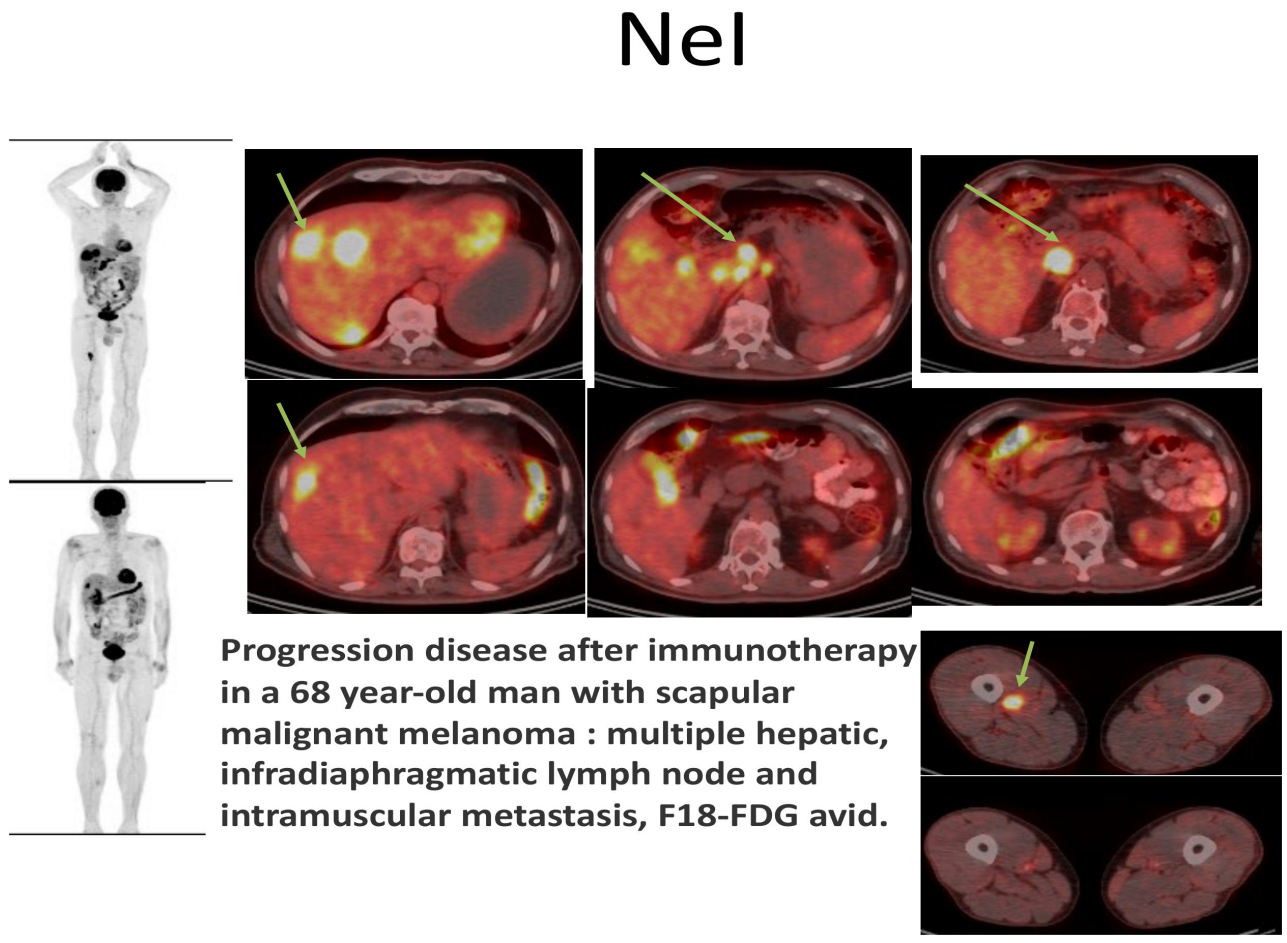
Malignant melanoma and various aspects of metastatic spread, as identified and depicted by PET-CT.

In 1992, the first MR appearance of intra-abdominal metastatic melanoma as being similar to melanoma metastatic to other sites was reported [40]. Research [41] in 1993 described the sensitivity of PET-CT as 100% in the detection of intra-abdominal and lymph node metastases, and, afterwards, in 2000, research continued with a study [42] that had, as an objective, to compare staging by whole-body positron emission tomography using fluorine-18 fluorodeoxyglucose (18F-FDG) to staging by conventional methods. This study concluded that 18F-FDG PET is a more sensitive method in the detection of widespread metastases from malignant melanoma than conventional methods (had been), a conclusion also found in other research [43]. The finding enables the avoidance of ineffective mutilating surgery and concludes that 18F-FDG PET is a useful adjunct to clinical examination in the staging of melanoma. The virtue of PET-CT in detecting metastases from malignant melanoma is illustrated in Figure 3. Moreover, FDG PET/CT can be used in order to assess treatment response [44] by evaluating the outcome as influenced by immunotherapy. When looking [45] at the compared efficacy of ultrasound, MRI, CT scan, and PET-CT scan, PET-CT exam registered the best results, both of which concern staging and re-staging of the disease. The importance of sentinel lymph node and proper identification of the lymphatic drainage in cutaneous melanoma was also analyzed and studied intensely, without definitive conclusions being reached, as also shown in Figure 2 [46].

More recently [47], the uptake of fluorodeoxyglucose (FDG) by bone marrow (BM) and adipose tissue was researched because the two sites are known to reflect the systemic inflammatory response to cancer cells. The goal of this study was to evaluate the prognostic value of F-18 FDG uptake in malignant melanoma (MM) and to characterize visceral adipose tissue (VAT) and subcutaneous adipose tissue (SAT) on PET/CT images. The conclusion was that CT HU, SUV mean of SAT and VAT, and BLR provide information regarding the prognosis of DPFS (prediction of disease progression) in malignant melanoma.

Endoscopic ultrasound-guided fine-needle aspiration EUS-FNA for pancreatic lesions can be used in the diagnosis of melanomas if the tumor can be reached through this method [48,49].

### 3.4. Special Discussions: Melanoma of Unknown Primary, Achromic/Amelanotic Melanoma, Melanoma of the Lower Genital Tract, and Melanoma of the Urinary Tract

#### 3.4.1. Melanoma of Unknown Primary

Melanoma of unknown primary (MUP) is defined as a metastatic melanoma within the lymph nodes, subcutaneous tissues, and other distant sites without an evident primary lesion and its incidence is low (3.2%) among all the cases of melanoma, as shown by [50]. It was reported [51] that it comprises 3–4% of all melanomas and that, based on the evidence that melanoma can undergo regression at its primary site, spontaneous regression of the primary lesion is a well-established theory. MUP and stage-matched, known-primary-site melanoma (MKP) share similar prognostic factors.

In a case series of 15 cases with visceral metastases from melanoma [52], it was found that in eight instances, the primary was unknown, whereas, in seven instances, it was known. Among 15 patients, 10 were men and 5 were women. All metastases were found in the abdominal cavity: (liver (3), abdominal lymph nodes (4), stomach (2), bowel (4), omentum (1), spleen (1), esophagus (1), and adrenal (2)). In one instance, the metastatic deposit was in the brain, and in another, it was in a vertebral body. In six instances, visceral metastases were found in multiple sites.

It was mentioned [53] that between 4 and 9 percent of cases of gastrointestinal melanoma have unidentified primary tumors. More so, rapid identification and resection of gastrointestinal melanoma could increase patient survival rates and prevent complications such as intestinal obstructions.

On the subject of the differential between a primary hepatic melanoma or melanoma of an unknown primary, literature research describes [54] that, rarely as melanoma is diagnosed in visceral organs as a primary lesion, suspected primary hepatic melanoma is extremely rare and has only scarcely been shown in a handful of case reports. Similar observations (difficult differential between a primary and metastatic lesion) were found in the case of the colon [55]. The same research states that the actual existence of primary melanoma in the gastrointestinal tract, outside the esophagus or the anorectum, actually exists, although contested, and the proof shown is a case-presentation of a malignant melanoma of the caecum.

It is uncommon for a melanoma of unknown primary origin to infiltrate the liver in a diffuse fashion and the report demonstrates the difficulty of making a noninvasive diagnosis of metastatic melanoma with diffuse hepatic infiltration [56]. Furthermore, there are studies that illustrate treatment approaches in the case of a melanoma of unknown primary [57], describing cytotoxic T-lymphocyte-associated antigen 4 (CTLA-4, ipilimumab) and programmed death protein-1 (PD-1) antibodies (PD-1, nivolumab), with a poor prognosis (4-months’ survival).

Moreover, metastatic malignant melanoma may mimic ovarian tumors with occult or regressed primary tumors [58]. More so, it may be difficult to differentiate ovarian melanoma from epithelial ovarian malignancies using standard pre-operative imaging modalities (e.g., CT, MRI) and histopathologic examination of frozen sections. Immunohistochemistry could provide a conclusive diagnosis.

#### 3.4.2. Achromic/Amelanotic Melanoma

Survival outcomes following a diagnosis of an achromic melanoma are worse than that following diagnosis of a pigmented form [59,60].

The presence of mitoses in amelanotic melanoma, regardless of Breslow thickness or other clinicopathologic features, suggests that amelanotic melanomas may also grow faster than pigmented melanomas.

#### 3.4.3. Melanoma of the Lower Genital Tract

Cancers that originate from the gynecological tract are uncommon and aggressive [61]. The vulva is the most common location (70%), followed by the vagina and, less frequently, the cervix. The clinical outcome of patients with melanoma of the female genital tract is very poor, with a 5-year overall survival (OS) of 37–50% for vulvar, 13–32% for vaginal, and approximately 10% for cervical melanoma. A vaginal location of a malignant melanoma has been described to have a poorer prognosis than a vulvar lesion [62]. The aspect according to which there can also appear metastases to the genital organs (for instance to the endometrium) from an extra-abdominal primary site, such as the brain, was also described in medical literature [63], among other similar few cases.

#### 3.4.4. Melanoma of the Urinary Tract/Apparatus

When a tumor manifests in an unusual location, it is more difficult to diagnose [64]. Due to their rarity in the urinary tract, primary melanomas, carcinoid tumors, and epithelioid angiosarcoma may pose diagnostic difficulties.

Historically, uroscopists have believed that the color of urine provided the most important diagnostic clues. In modern medicine, urine color can still provide some diagnostic information. Pigmented cells are a rare and unexpected finding in urine cytology, and they can simultaneously provide important diagnostic clues or represent a hazard. The significance of pigmented cells in urine cytology has been studied and discussed [65]. The principal differential diagnosis for cytoplasmic pigmented granules may include hemosiderin, lipofuscin, and melanin, each of which has a distinct pathogenesis and clinical significance.

##### The Prostate Gland

In a systematic review presented in 2021 [66], it was found that from a total of 25 studies describing 45 cases, the majority of prostate cancer cases were metastases, with only 10 cases of primary prostate cancer. With a wide range, the median age of patients was 61 years, and 89% were symptomatic at presentation, most commonly with obstructive symptoms (83%). Histopathological analysis and frequently immunohistochemistry are required for diagnosis. Metastatic melanoma of the prostate has a dismal prognosis, with a median overall survival of three months; in contrast, 29 percent of patients with primary prostatic melanomas reported in the medical literature survived for more than five years.

##### The Urinary Bladder

In a review looking at the malignant non-urothelial neoplasms of the urinary bladder, it was found that non-urothelial bladder tumors frequently pose diagnostic and therapeutic difficulties, and that primary non-urothelial bladder tumors are uncommon in the European and North American geographical areas, accounting for less than 5 percent of all bladder lesions [67].

Squamous cell carcinoma, adenocarcinoma, small cell carcinoma, sarcoma and carcinosarcoma/sarcomatoid tumors share an unfavorable prognosis, despite aggressive surgical management that relates both to an aggressive biological behavior. In addition to paraganglioma, primary melanoma, and lymphoma, extremely rare bladder tumors include paraganglioma. It has been reported in a review of the literature [68] that less than 0.2% of all melanomas are primary melanomas of the genitourinary tract.

In [69], it was noted that most urinary bladder metastases originate from breast carcinoma and skin melanoma. Regardless of whether it is a primary or secondary lesion of malignant melanoma, the tissue examination yields the same results.

Immunohistochemistry aids in accurate diagnosis, but the distinction between primary and metastatic tumors remains an important issue. Typically, bladder melanoma is aggressive and fatal.

The prognosis for patients with primary bladder melanoma is dismal, and the treatment of this rare condition presents a therapeutic challenge [70]. In the same author’s experience, a multidisciplinary approach is required for the diagnosis and treatment of this uncommon cancer.

Immune checkpoint inhibitors (ICIs) have substantially altered the treatment of urological tumors, for which multiple agents are currently approved. Nevertheless, the majority of patients discontinue treatment because of disease progression or the onset of severe immune-related adverse events (IRAEs). Following promising results in patients with melanoma, retreatment with an ICI is becoming an increasingly attractive option for certain patients.

A literature review [71] focusing on the feasibility, safety, timing, and efficacy of ICI rechallenge in genitourinary cancers showed there is limited data. The study categorized the various ICI retreatment strategies into three main clinical scenarios: retreatment after discontinuing a prior course of ICI while on response; retreatment after an interruption due to IRAEs; and retreatment after progression while on ICI therapy. The advantages and disadvantages of these options in the field of urological tumors are then discussed, and different ideas for the improvement of future clinical trials are provided. Similar research was conducted regarding the possibility of treatment of urothelial and non-urothelial bladder cancers with immunotherapy [72].

Recently, oncolytic virus therapy has been recognized as a promising new cancer treatment option [73]. Oncolytic viruses replicate only in cancer cells, killing them without harming healthy cells. Notably, T-VEC (talimogene laherparepvec, formerly known as OncoVEX GM-CSF), an oncolytic herpes simplex virus type 1, was acknowledged and approved by the FDA (U.S. Food and Drug Administration) in October 2015 for the treatment of inoperable melanoma, and was later approved in Europe and Australia in 2016. During the past decade, numerous types of oncolytic viruses’ efficacy against urological cancers has been investigated in preclinical studies, and some have already been evaluated in clinical trials. In 2016, for instance, a phase I trial of the third-generation oncolytic Herpes simplex virus type 1 G47 in prostate cancer patients was concluded.

##### The Urethra

Less than 200 cases of urethral melanomas have been reported in the scientific literature, with MM of the female urethra accounting for less than 0.2% of all primary melanomas [74]. Due to the delayed presentation, early onset of metastasis, and aggressive tumor biology, the prognosis for multiple myeloma (MM) has remained generally poor despite adequate local control. The main treatment is surgery, with adjuvant radiation contributing to local control but not to overall survival. Options for chemotherapy and immunotherapy are being investigated in both adjuvant and palliative settings.

Medical literature describes primary melanomas of the vagina with urethral invasion [75], and a mass in the urethral meatus and hematuria are frequent clinical manifestations [76].

##### The Ureter

Ureter-originating malignant melanoma is considered to be extremely uncommon [77]. Genetic variants associated with the increased disease risk have not yet been investigated. In tumor samples, we identified 38 somatic single nucleotide variants and 9 somatic insertions and deletions (INDELs). The Cancer Gene Census database was used to identify seven predisposing genes and two driver mutation genes. The study is pioneer in providing evidence that the distinct phenotypes of primary malignant melanoma of the ureter may result from different genetic variations. A primary lesion of a malignant melanoma of the urinary tract was associated with a worse prognosis than primary urothelial carcinoma of the urinary tract.

### 3.5. Melanoma at the Level of the Umbilicus

Being situated next to intra-abdominal and different pelvic anatomic structures, the skin surrounding the umbilicus is distinctive. In addition to cutaneous primary malignancies, this site is frequently affected by metastatic disease. In 1988 [78], clinical and pathologic characteristics of 77 umbilical malignancies diagnosed in one single institution were presented. Eighty-eight percent of cancers began outside of the umbilicus, and twelve percent were primary skin tumors. Primary melanoma of the umbilicus is extremely uncommon, there are few published data on it (27 cases from 1916 to 2018, [79]), and its incidence is unknown.

### 3.6. Particularities of Surgical Techniques and Other Treatment Options

Malignant melanoma is a disease whose progression is unpredictable [80]. Malignant melanoma is a disease whose progression is unpredictable. Cancers detected in stage I and II have a high chance of being cured if they are appropriately treated: excisional biopsy with safety margins proportional to tumor thickness. Lymphoscintigraphy with sentinel node identification and biopsy became mandatory for staging malignant melanoma. Increasingly sophisticated techniques (RT-PCR) are applied to the sentinel lymph node in order to detect isolated tumor cells, whose clinical significance is currently under debate. The occurrence of metastases is a dramatic phenomenon because chemotherapy, radiotherapy, and biologic therapy are mostly barely effective. Surgery is the only treatment that can prolong the survival of selected patient groups in this situation. More than that, there are authors [81] who underline the importance of the sentinel lymph node technique in order to ensure a precise diagnosis as a stage II patient with melanoma can also develop another misleading process, such as a lymphoma causing lymphadenopathy.

According to the particular tumor location, surgical options are hereby mentioned briefly in the case of primary melanomas. Research [82] has revealed that esophageal resectable primary malignant melanoma is optimally treated with surgery and that, if surgery is not possible in order to treat dysphagia, endoscopic therapy should be considered. Metal stents in the distal esophagus for palliation were also employed with good results. The main surgical option in primary gastric cancer is partial gastrectomy, performed in around half of the cases, with cuneiform resection and total gastrectomy in the second and third places. In properly selected patients, pancreatic resection for locally advanced nonpancreatic or recurrent intra-abdominal malignancies is feasible [83]. The capacity to obtain disease-free margins via en-bloc resection is a crucial aspect of treatment in primary pancreatic melanoma. Colorectal and anal melanoma may have the following surgical options in correlation with the precise tumor site, tumor size and local expansion: right or left hemicolectomy, transverse colectomy, anterior resection, Hartmann’s procedure, abdomino–perineal resection, or even colostomy in locally advanced tumors. Ovarian primary melanoma, associated or not with other ovarian lesions (for instance, ovarian teratoma) also meets surgery as the current standard of care [84]. In the majority of previous studies, the 5-year survival rate for patients with anorectal malignant melanoma (ARMM) was less than 20%. The optimal surgical treatment has remained contentious. A retrospective study [85] aims to evaluate the prognosis of ARMM patients who underwent curative surgical resection and finds that the majority of patients eventually succumb to the disease, regardless of treatment. Depending on the subset of patients selected, both APR and WLE play significant roles in the management. When possible, local treatment should be preferred. Abdomino–perineal resection should be performed in cases of nodal disease or recurrence. Surgical tumor resection remains the mainly preferred treatment approach also for small bowel primary melanomas.

Long-term survival is uncommon in metastatic melanoma to the pancreas, so it appears that surgical resection is only a palliative procedure, despite the limited experience [86]. Even if there is no effective systemic therapy, surgery may be considered as part of an aggressive multidisciplinary approach in certain cases of malignant melanoma that has spread to a single or to multiple visceral sites.

The case of a robotically extended ultrasound-guided distal pancreatectomy for pancreatic metastases from uveal melanoma has also been described [87]. Patients with pancreatic metastases (of both cutaneous and ocular origin) who underwent resection exhibited a significant survival advantage over those treated non-surgically. The popularity of minimally invasive pancreatectomy is growing. While maintaining the oncological tenets of resection, minimal postoperative morbidity and earlier return to daily activities confer distinct advantages over conventional surgery in certain patients. Recent reports suggest that the use of robots may offer some advantages over conventional laparoscopy, particularly for patients who require technically difficult surgeries. These benefits relate primarily to conversion rate, length of postoperative hospital stay, andthe number of cases required to overcome the learning curve and achieve optimal performance.

Resection of resistant adrenal metastases has been facilitated by minimally invasive adrenalectomy [88]. Despite systemic therapy, the adrenal gland may function as a sanctuary for metastatic growth. The purpose of the study was to assess the efficacy of minimally invasive selective adrenalectomy during immunotherapy. Even though an increase in objective durable response to immunotherapies and targeted treatments in metastatic melanoma has been noted, minimally invasive adrenalectomy is advantageous in cases of adrenal disease resistant to medical treatment.

If necessary (the metastatic malignant melanoma disease involves various organs), multiorgan abdominal resections including the ovary, jejunum, stomach, and pancreas can be indicated [89], with a potential to offer a survival benefit.

The median survival time for patients with distant melanoma metastases is between 4 and 8 months [90]. Previous research has demonstrated that complete resection of pulmonary and hollow viscus gastrointestinal metastases improves survival. The authors hypothesized that patients with metastatic disease to solid intra-abdominal organs might also benefit from total surgical resection. The conclusion was that, in highly selected patients with intra-abdominal solid organ metastatic melanoma, aggressive attempts at complete surgical resection may improve OS. It is crucial that the number of metastatic sites after complete resection does not appear to affect overall survival. In a study published in 2007 [91], personal experience in determining the role of resectional surgery in metastatic melanoma to the abdomen was discussed. The conclusion was that, in a highly selected group of patients with intra-abdominal melanoma metastases, the resection of intra-abdominal metastases with curative intent was associated with longer survival than palliative resection [92,93]. Moreover, those who underwent palliative resection experienced significant symptom relief with little morbidity [94]. In addition to that, patients selected for surgical resection after checkpoint blockade have a relatively favorable prognosis, particularly if they responded to immunotherapy and undergo total resection of isolated progressing or responding disease [95].

Evidence regarding the efficacy of curative metastasectomy (CM) for patients with malignant melanoma (MM) is limited, particularly in the era of effective systemic therapy [96]. In patients with MM, a systematic review and meta-analysis were conducted to determine the role of CM in comparison to incomplete or nonsurgical treatment. Forty studies involving 31,282 patients (CM, 9958; non-CM, 21,324) were considered for the final analysis. CM was associated with a significantly lower risk of death compared to the absence of CM. Analysis of subgroups revealed that the outcome was independent of the efficacy of systemic treatment and the anatomical location of metastases. Increasing age, elevated lactate dehydrogenase (LDH), male gender, prior stage 3 disease, multiple metastases and organ sites, and shorter disease-free interval were associated with a poor prognosis. LDH and S100B have been found to be reliable serum biomarkers in late-stage melanoma [97]. Curative metastasectomy for malignant melanoma is associated with a lower mortality risk than noncurative treatment methods. Whenever technically feasible, CM should be included in the multimodal treatment of MM.

Metastases from an unknown primary to the small bowel can well be a cause of obstruction and manifest in an emergency setting [98,99]: a description of bifocal metastases of melanoma treated through partial resection of the jejunum and distal ileum with termino-terminal anastomosis have been described.

Followed by adjuvant therapy, radical surgery constitutes the most rational method of treatment in the situation of primary prostatic melanoma, as well.

Pancreatic resection, apart from the situation of a secondary melanoma, can also be indicated in the clinical setting of an unknown primary lesion [100].

Due to the tumor’s occult or rapid metastasis to extra-abdominal sites, intra-abdominal debulking surgery would not prolong the survival of patients with metastatic ovarian melanoma.

In a systematic review looking at the effectiveness of yttrium-90 radioembolization in the treatment of unresectable liver metastases from melanoma, the conclusion was that, with encouraging effects on disease control and survival, 90Y radioembolization is a promising alternative therapy for the treatment of liver metastases of melanoma that cannot be resected [101].

Additional information and correlation between anatomical sites, a few examples of influential genes, and various response rates to immunotherapy can be found in Table 3.

### 3.7. Presenting in an Emergency Setting

Visceral metastases from malignant melanoma (stage M1c) confer a very poor prognosis [120], as documented in the most recent revised version of the TNM/AJCC staging system. It is uncommon for intra-abdominal complications to necessitate emergency abdominal surgery. In an effort to improve survival for melanoma patients with visceral disease, elective curative surgery combined with novel cytotoxic systemic therapies is under investigation, as reported for the year 2014.

One of the many facets of an emergency situation is bowel perforation [121], which imposes enterectomy.

Malignant biliary obstruction is typically caused by primary malignancies of the pancreatic head, bile duct, gallbladder, liver, and Vater’s ampulla [122]. Lesions that have spread from other primary sites to these organs or nearby lymph nodes are a less common cause of biliary obstruction. Renal cancer, lung cancer, gastric cancer, colorectal cancer, breast cancer, lymphoma, and melanoma are the most prevalent primary cancers. They may be challenging to distinguish from primary hepato–pancreato–biliary cancer based on imaging studies or even biopsies [123]. In this regard, peritoneal metastases from extra-abdominal cancer, in a population-based study from 2018 [124], peritoneal metastases (PM) were typically a symptom of intra-abdominal cancers, such as colorectal or ovarian cancer. However, extra-abdominal cancers can also spread to the peritoneum. This is the first population-based study to report the incidence of extra-abdominal cancer-related pulmonary metastases. The three most prevalent primary cancers were melanoma, breast, and lung. Consistently, a poor prognosis accompanied peritoneal disease metastases.

An important differential has to be made with intra-abdominal metastases of soft tissue sarcoma [125], which can present with common manifestations, such as intestinal obstruction, abdominal pain, mass, gastrointestinal bleeding, and abdominal distension. GIST tumors can also appear as melanomas, and sometimes, only immunohistochemistry can diagnose the proliferation correctly [126,127,128]. Another mimicker for melanoma is a malignant neuroectodermal tumor with a junctional component [129], identified recently as a rare and aggressive tumor that typically develops in the small intestine of adult patients.

The rare situation of an intussusception in an adult patient can also be caused by an intraperitoneal metastasis from a melanoma or by a primary small bowel melanoma, and can manifest with acute peritonitis, hemorrhage, and obstruction [130].

Long-term survival is only associated with complete resection of all metastases; debulking should not be attempted [131].

Medical literature reports severe side effects of treatment in the case of malignant melanoma, which had metastasized to the abdominal cavity. As a cause of that, patients can also present in emergency settings. The occurrence of the acute tumor lysis syndrome following encorafenib and binimetinib for v600E metastatic melanoma in the case of a large intra-abdominal mass, has been described [132,133]. Another emergency setting is that of a spontaneous splenic rupture in a patient with metastatic melanoma treated with vemurafenib [134]. Due to the intraoperative finding of hemoperitoneum caused by a two-step splenic rupture, a splenectomy was performed. In the absence of metastases, histopathology confirmed a splenic hematoma and capsule laceration. A similar phenomenon (of rupture associated with vemurafenib treatment) can occur in the case of the liver [135]. The rare possibility of immunotherapy causing non-inflammatory bowel perforations as a result of rapid tumor regression, is an important aspect of the medical literature [136,137], as the toxicity of immune checkpoint inhibitors like ipilimumab and nivolumab is related to their clinical efficacy. Adjuvant high-dose ipilimumab administered after ipilimumab and nivolumab for inoperable metastatic disease [138] were also reported.

In patients with cutaneous melanoma, abdominal manifestations include both involvement due to metastatic spread and immune checkpoint inhibitor-induced adverse events [139]. CT is useful for identifying colitis, enteritis, and pancreatitis, whereas MRI is essential for identifying autoimmune pancreatitis. Imaging appearances following immunotherapy, including adverse events, are distinctive and perplexing at times [140]. Moreover, imaging is integral to managing patients receiving immunotherapeutic agents, and a comprehensive understanding of its mechanism, response patterns, and adverse events is essential for accurately interpreting imaging studies. Immune-related adverse events are a new class of side effects caused by abundant upregulation (IRAEs) [141], furthermore, the author explains that it is compulsory for the practicing radiologist to be able to recognize these events in order to optimally contribute to the care of patients receiving immunotherapy with checkpoint inhibitors [142,143].

## 4. Prognosis

Despite recent advances in treating solid cancers, particularly the success of immunomodulatory antibody therapies for a variety of cancer types, many patients continue to fail to respond to treatment [144].

It is therefore highly important to correctly identify biomarkers predicting clinical responses to treatment and patient survival, which would not only aid in targeting treatments to patients most likely to benefit but also provide mechanistic insights into the reasons for the therapy’s success or failure.

The employment and use of peripheral blood (“liquid biopsy”) in the diagnosis process offers numerous benefits not only for predicting treatment responses at baseline, but also for monitoring patients undergoing treatment.

Assessment of the tumor microenvironment and immune cells infiltrating the tumor also provides important information on cancer-host interactions; however, the need for tumor tissue makes this more difficult, particularly for monitoring sequential changes in a single patient.

In a review and meta-analysis of the evidence on circulating tumor DNA (ctDNA) levels, those were correlated with melanoma patient survival [145]. The conclusion was that ctDNA is a powerful prognostic biomarker for patients with advanced-stage melanoma, robust across tumor (e.g., genomic profile) and patient (e.g., systemic therapy) characteristics.

MiRNAs’ diagnostic and prognostic value in cutaneous melanoma (CM) has been extensively studied and is supported by sophisticated bioinformatics tools. From early studies utilizing miRNA arrays with several limitations to the most recent NGS-derived miRNA expression profiles, an accurate diagnostic panel of a comprehensive pre-specified set of miRNAs that could aid in the timely identification of specific cancer stages remains elusive, primarily due to the heterogeneity of the approaches and samples. An analysis of the correlation between specific miRNA expression profiles and the expression signatures of known gene targets was performed using publicly available NGS data [146]. Using network analytics and machine learning, we developed non-linear classification models capable of accurately predicting CM recurrence and metastasis based on two newly identified miRNA signatures. Subsequent unbiased analyses and independent test sets (i.e., a dataset not used for training, as a validation cohort) using our prediction models yielded 73.85% and 82.09% accuracy, respectively, in predicting CM recurrence and metastasis.

More information regarding the clinical trials available that could potentially modify the prognosis with their results, can be found in Table 4.

## 5. Discussion

Typically, malignant gastrointestinal melanoma is a metastatic tumor. It is extremely rare for malignant melanoma to originate in the digestive tract. Primary small bowel MM appears to be a fairly uncommon illness that doctors should be better aware of in order to develop more precise strategies for early diagnosis and treatment. Surgical tumor excision remains the most desired therapeutic method, and multi-organ abdominal resections, including the ovary, jejunum, stomach, and pancreas, have been documented for use as necessary.

The outcome was not directly related to systemic treatment and the anatomical location of metastases, as shown by subgroup analysis. Abdominal symptoms in patients with cutaneous melanoma can be related to metastatic dissemination and immune checkpoint inhibitor-induced adverse effects.

As there is no consensus regarding surveillance, staging, and treatment of disseminated disease, the authors believe that a meta-analysis of intra-abdominal melanoma with its various forms and peculiarities in terms of diagnostic and treatment issues and challenges could help clarify the optimal patient-specific approach.

## Figures and Tables

**Table 1:** Databases, search terms, and filters used in documenting the present literature review on intra-abdominal malignant melanoma, accessed between 3 May 2022 and 3 June 2022.

Research Site/Database	Search Terms	Additional Filters
www.scopus.com	”intra-abdominal metastases from malignant melanoma”	“medicine”, “review”, “English”
www.scopus.com	”intra-abdominal malignant melanoma”	2018–2022, subject area “medicine”, “English”
www.scopus.com	”melanoma of unknown primary”, and “abdominal AND melanoma AND systematic AND review”	“English”, “medicine” and “journal”, “after 2015”
www.scopus.com	“melanoma of the urinary tract review”	“medicine”, 2017–2022, “English”, “Journals”, “reviews”
www.pubmed.org	“intra-abdominal malignant melanoma”	“English” and “humans”
www.sciencedirect.com	“intra-abdominal malignant melanoma”	“subscribed journals”, 2017–2022, review articles, medicine and dentistry
www.sciencedirect.com	“amelanotic melanoma of the abdomen”,	“subscribed journal”, 2017–2022, “medicine and dentistry”
www.sciencedirect.com	“achromic AND melanoma AND metastases”	
www.sciencedirect.com www.pubmed.org http://academic.oup.com	“(urinary OR kidney OR bladder OR urethra OR ureter) AND melanoma”	
http://academic.oup.com	“intra-abdominal malignant melanoma”	“journal article”, 2017–2022, medicine and health

**Table 2:** Examples of different research illustrating various assets of the study of melanoma in animal models.

First Author of the Study and Year of Publication	Type of Model	Principle and Assets
[7], 2004	Murine model	It may help in the study of lymph node metastases
[8], 2021	Murine model	It can detect early the appearance of metastases and can monitor the response to immune checkpoint inhibitors
[9], 1997	Murine model	Allows experimental local treatment of the melanoma
[10], 2014	Murine model	It allows the study of the anti-proliferative effect of mushroom mycelia, including in comparison with chemotherapy regimens
[11], 2018	Patient-derived orthotopic mouse models	It may show how melanoma cell lines implanted in the abdominal cavity can form nodules
[12], 1992	Murine model	Radio-immunotargetting-monoclonal antibodies in intraperitoneal malignancy
[13], 2014	Murine model	It allows the study of the pattern of fat loss in cancer
[14], 2017	Murine models	It allows the study of perioperative events that influence cancer recurrence risk
[15], 2022	Review of ultrasound and microbubble-mediated delivery on animal models	The combination of ultrasound and microbubbles is a promising strategy for increasing vascular permeability, thereby enhancing drug delivery to tissues. This combination has also been applied to gene and protein delivery, including immunotherapy cytokines and antigens.

**Table 3:** The anatomical site, genes involved, and response to immunotherapy.

Primary Site	Genetic Factors Determining the Anatomic Location	Data on Response to Immunotherapy
Predisposition to uveal and cutaneous melanoma	BAP1 syndrome	BAP1 syndrome has many facets as a complex cancer syndrome characterized by an increased risk of rare malignant mesothelioma, malignant skin and uveal melanoma, spitzoid-type skin lesions, and other tumors.
		Detection of this syndrome is crucial for the survival of individuals at high risk [102].
	Amplification of mutated NRAS	The amplification of mutated NRAS is associated with Congenital melanoma in neurocutaneous melanocytosis [103]
	CDKN2A germline mutation	Hereditary pancreatic carcinoma shows extant phenotypic and genotypic heterogeneity as evidenced by its integral association with a variety of hereditary cancer syndromes inclusive of the familial atypical multiple mole melanoma (FAMMM) syndrome in concert with CDKN2A (p16) germline mutations [104].
	Variation in the melanocortin-1receptor MC1R gene	The results of a study by [105] suggest that inherited variation in MC1R may play a significant role in the anatomic site presentation of melanomas and may vary in relation to skin pigmentation phenotype.
Tumor immune microenvironment	IFN response-related gene signature (UBE2L6, PARP14, IFIH1, IRF2, and GBP4)	A novel five-IFN response-related gene signature (UBE2L6, PARP14, IFIH1, IRF2, and GBP4) was developed, which provided a better and more comprehensive understanding of the tumor immune landscape and demonstrated excellent performance in predicting patient outcomes for SKCM (skin cutaneous melanoma) [106].
Mucosal melanomas (respiratory, gastrointestinal, and of the urogenital tract)	MM is genetically distinct from its skin-based counterparts. Common drivers in cutaneous melanoma, such as B-raf proto-oncogene serine/threonine kinase (BRAF), have a lower mutation rate in multiple myeloma (MM), whereas mutations of other genes, such as the KIT proto-oncogene, receptor tyrosine kinase (KIT), and splicing factor 3b subunit 1 gene (SF3B1), are more prevalent The presence of KIT mutations, which are potential targets of tyrosine kinase inhibitors currently in clinical trials (imatinib), as well as SF3B1 mutations, CDK4 amplifications, and CDKN2A gene deletions are being investigated in clinical trials. MM of the ovaries related to KIT gene mutation and loss of heterozygosity of the PTEN region MM of the vagina related to downregulation of the following 4 genes: STATH, EEF1A2, TTR, and CDH2.	Immune checkpoint inhibitors of CTLA4 (ipilimumab) and PD-1 were administered to the patient (pembrolizumab and nivolumab). Research [107] points to the fact that LOH (loss of heterozygosity) of the PTEN region is one of the molecular alterations of an ovarian mature cystic teratoma, and a KIT mutation is an additional event that promotes the oncogenesis of a melanoma arising from an ovarian mature cystic teratoma. The results of this case study suggest that itraconazole may be an effective treatment option for vaginal primary malignant melanoma. In addition, the authors identified potential itraconazole target genes, which could aid in the elucidation of the disease’s underlying mechanism and the development of new therapeutic agents [108].
Primary esophageal	C-KIT, PDGFR, NRAS, KRAS mutations NF1 was the gene most frequently altered. Other mutated genes included SF3B1, KRAS, BRCA2, KIT, and TP53.	
Stage IV melanoma	Compared to primary disease, metastatic disease is enriched for MDM2 and MDM4 amplifications, and amplifications are associated with decreased overall survival. Amplifications of MDM2/4 are associated with a higher incidence of brain and liver metastasis. USP7 and PPM1D, two negative regulators of p53, are also altered in metastatic melanoma relative to primary disease. SKI pathways inducing progression of melanoma [109]. Pembrolizumab initially appeared to be significantly less effective in melanoma and non-small cell lung cancer (NSCLC) patients with liver metastases.	[110]: In a study comprising 14,433 patients with stage IV melanoma has found that Immunotherapy was distributed unequally among patients with stage IV melanoma. The rates of surgical resection of metastatic disease for stage IV melanoma did not differ between the checkpoint inhibitor era and the pre-checkpoint inhibitor era across all facilities. Patients with melanoma or GBM and amplifications in MDM2/4 and CDKN2A alterations may benefit from combinations of targeted inhibitors of MDM2/4 and CDK4/6, as well as immunotherapy, according to the authors [109]. SKI plays additional roles both within and without the nucleus. In normal melanocytes and primary non-invasive melanomas, SKI is predominantly nuclear, whereas, in primary invasive melanomas, SKI is both nuclear and cytoplasmic. SKI distribution is intriguingly nuclear and cytoplasmic or predominantly cytoplasmic in metastatic melanoma tumors [111]. Case reports [112,57] showed a durable response to anti CTLA-4 and anti-PD1. Several recent clinical and translational studies [113] have focused on the impact of liver metastases on the effectiveness of immune checkpoint inhibitors in patients with solid-tumor malignancies. A retrospective study on 20 consecutive small bowel melanoma metastases was described. The conclusion was that although medical treatments for metastatic melanoma have dramatically improved survival, surgical control of life-threatening localizations such as small bowel metastases is frequently a prerequisite for long survival [114]. Compared to metastases removed prior to ipilimumab therapy, post-treatment lesions exhibited significantly lower HL class I expression on melanoma cells; HLA class I downregulation was most pronounced in metastases from nonresponding patients that were progressing. The results suggest that HLA class I downregulation may serve as a mechanism of ICI resistance [115]. Case report [116] described immunotherapy with ipilimumab and pembrolizumab.
Prediction factors that can influence the response to immunotherapy: 1. CT texture analysis 2. Anatomic location 3. The evaluation of PD-L1 immunohistochemical expression		The conclusion of a study [117] was that patients with metastatic SM may use CT texture analysis-derived tumor skewness and variation of entropy between baseline and first control CT examination as predictors of favorable response to anti-PD1 monoclonal antibodies. In a multivariate analysis, patients with lung metastases had superior ORR and progression-free survival, whereas patients with liver metastases had inferior ORR and progression-free survival, demonstrating that treatment response and, consequently, survival can vary with anatomic location [118]. An atlas of PD-L1 for pathologists has been created [119].

**Table 4:** Examples of current clinical trials on melanoma. More information on current trials on melanoma can be found at: www.mayo.edu, www.curemelanoma.org and www.clinicaltrials.gov.

(1) A study comparing Temozolomide and Selumetinib in metastatic melanoma of the eye	Interventional, phase 2, developed in Rochester, Minn, NCT ID: NCT01143402
(2) Carbozantinib S Malate compared with Temozolomide in treating patients with melanoma of the eye	Interventional, phase 2, developed in Rochester, Minn, NCT ID: NCT01835145
(3) Dasatinib in treating patients with locally advanced or metastatic mucosal melanoma, acral melanoma, vulvovaginal melanoma, that cannot be removed by surgery	Interventional, phase 2, developed in Rochester, Minn, NCT ID: NCT00700882
(4) A study of the effectiveness of stress management therapy for patients with melanoma	Interventional, Rochester, Minn, Site IRB Rochester, Minnesota: 14-001651
(5) A study comparing vaccines with or without an autologous tumor lysate to treat stage III or IV melanoma to prevent a recurrence	Interventional, phase 2, Scottsdale/Phoenix, Ariz, and Rochester, Minn NCT ID: NCT02301611
(6) A Study to Describe Patterns of Treatment, Demographics, Clinical Characteristics, and Overall Survival in Patients with Unresectable or Metastatic Melanoma	Observational NCT ID: NCT02780089
(7) MART-1 Antigen with or Without TLR4 Agonist GLA-SE in Treating Patients With Stage II-IV Melanoma That Has Been Removed by Surgery	Interventional NCT ID: NCT02320305
(8) A Study to Evaluate the Safety and Effectiveness of Intratumoral and Intravenous Injection of Vesicular Stomatitis Virus Expressing Human Interferon Beta, and Tyrosinase Related Protein 1 (VSV-IFNb-TYRP1) in Patients with Metastatic Ocular Melanoma and Previously Treated Patients with Unresectable Stage III/IV Cutaneous Melanoma	Interventional, phase 1, NCT ID: NCT03865212
(9) Glembatumumab Vedotin in Treating Patients with Metastatic or Locally Recurrent Uveal Melanoma	Interventional, phase 2 NCT ID: NCT02363283
(10) Dendritic Cell Therapy After Cryosurgery in Combination with Pembrolizumab in Treating Patients with Stage III-IV Melanoma That Cannot Be Remove by Surgery	Interventional, phase ½, NCT ID: NCT03325101
(11) Surgery for Gastrointestinal Metastases of Malignant Melanoma—A Single Center Retrospective Cohort Study	Interventional, Sahlgrenska University HospitalGothenburg, Sweden NCT03879395
(12) Real World Study of Four PD-1 Agents in China	Interventional, The Affiliated Hospital of Qingdao UniversityQingdao, Shandong, China NCT03966456
(13) Nab-Paclitaxel and Bevacizumab in Treating Patients with Unresectable Stage IV Melanoma or Gynecological Cancers	Mayo Clinic Hospital in Arizona Phoenix, AZ, USA Mayo Clinic in Arizona Scottsdale, AZ, USA Mayo Clinic in Florida Jacksonville, FL, USA Mayo Clinic in Rochester Rochester, MN, USA NCT02020707

## Data Availability

Not applicable.
